# Chronological Registration of OCT and Autofluorescence Findings in CSCR: Two Distinct Patterns in Disease Course

**DOI:** 10.3390/diagnostics12081780

**Published:** 2022-07-22

**Authors:** Monty Santarossa, Ayse Tatli, Claus von der Burchard, Julia Andresen, Johann Roider, Heinz Handels, Reinhard Koch

**Affiliations:** 1 Department of Computer Science, Kiel University, 24118 Kiel, Germany; rk@informatik.uni-kiel.de; 2 Department of Ophthalmology, Kiel University, 24118 Kiel, Germany; Ayse.Tatli@uksh.de (A.T.); Claus.vonderBurchard@uksh.de (C.v.d.B.); Johann.Roider@uksh.de (J.R.); 3 Institute of Medical Informatics, University of Lübeck, 23562 Lübeck, Germany; j.andresen@uni-luebeck.de (J.A.); heinz.handels@uni-luebeck.de (H.H.)

**Keywords:** CSCR, OCT, fundus, autofluorescence, multimodal, registration, segmentation, deep learning, image analysis

## Abstract

Optical coherence tomography (OCT) and fundus autofluorescence (FAF) are important imaging modalities for the assessment and prognosis of central serous chorioretinopathy (CSCR). However, setting the findings from both into spatial and temporal contexts as desirable for disease analysis remains a challenge due to both modalities being captured in different perspectives: sparse three-dimensional (3D) cross sections for OCT and two-dimensional (2D) en face images for FAF. To bridge this gap, we propose a visualisation pipeline capable of projecting OCT labels to en face image modalities such as FAF. By mapping OCT B-scans onto the accompanying en face infrared (IR) image and then registering the IR image onto the FAF image by a neural network, we can directly compare OCT labels to other labels in the en face plane. We also present a U-Net inspired segmentation model to predict segmentations in unlabeled OCTs. Evaluations show that both our networks achieve high precision (0.853 Dice score and 0.913 Area under Curve). Furthermore, medical analysis performed on exemplary, chronologically arranged CSCR progressions of 12 patients visualized with our pipeline indicates that, on CSCR, two patterns emerge: subretinal fluid (SRF) in OCT preceding hyperfluorescence (HF) in FAF and vice versa.

## 1. Introduction

Central serous chorioretinopathy (CSCR) is an idiopathic chronic eye disease that affects mostly younger patients (20–50) [1] and can lead to severe bilateral visual acuity (VA) impairment. It is characterized by the accumulation of serous fluid at the posterior pole of the fundus, resulting in circumscribed detachment of the neurosensory retina and/or retinal pigment epithelium in the central retina (macula). It can be divided into acute CSCR, which typically lasts less than 6 months and is often spontaneously resolving, and chronic cases with multiple re-occurrences. This paper focuses on chronic CSCR, which often creates long-term VA loss [1,2,3].

Until today, no standardized treatment exists due to a very variable and overall poor outcome of all approaches. This creates a huge disease burden in the comparably young and often working patients, who often cannot be treated effectively and lose their ability to perform in their job, to drive a car or even to read. A better understanding of the disease, different subvarieties and their respective response to different treatment modalities might help to find more targeted and individualized therapies and could also help with individual disease course prognosis.

Although the pathogenesis is not fully understood, there is probably a diffuse dysfunction of the retinal pigment epithelium (RPE), the choroid or both structures. It is believed that as a result of the disruption of the autoregulation of choroidal perfusion, hypermeability of the choroid via a focal leak leads to RPE detachment. This RPE detachment then leads to mechanical damage that, as a result of a discontinuity of the RPE, ultimately results in a subretinal fluid accumulation with serous detachment of the neurosensory retina, which creates the VA impairment. Over longer periods, it can also create irreversible structural impairment and scarring [1].

Subretinal fluid (SRF) can be best and very precisely depicted in optical coherence tomography (OCT). In order to depict RPE alterations, fundus autofluorescence (FAF) is the most sensitive imaging method. While different biomarkers can be found in FAF patients, the most distinct biomarker in chronic CSCR patients is diffuse hyperfluorescence (HF) [4].

In contrast to SRF imaging in OCT, which is a structural and quantitative measurement of fluid accumulation and is very well understood, the underlying mechanisms that lead to HF formation and the meaning of HF in CSCR patients is only little understood. Likewise, the chronological relationship between these to biomarkers remains unclear: whether HF is a biomarker of altered RPE that starts leaking and thus precedes SRF formation and whether HF is caused by long-term SRF occurrence remain open questions.

**Figure 1:**
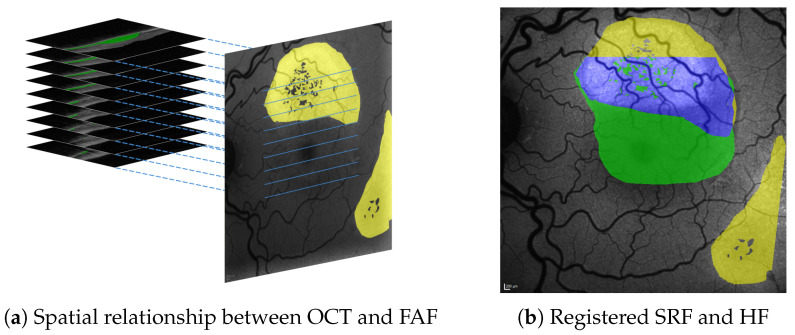
Spatial relationship between SRF (green) in 3D OCT B-scans and HF (yellow) in 2D en face FAF images (**a**), both of which are shown with roughly physically aligned orientations. Our approach allows to precisely register segmentations from both and visualise their overlap (shown in blue), e.g., in FAF image space (**b**).

One reason is that few comprehensive datasets of CSCR patients exist, given the drastically lower incidence (9.9/100,000 to 54.2/100,000 in men and 7.7/100,000 to 15.7/100,000 in women) [5,6] compared with widespread diseases such as age-related macular degeneration (AMD) (incidence 3500/100,000 over ages >50 years) [7]. Another reason, however, is that it requires two distinct imaging methods (OCT and FAF), which in turn give two completely different datasets (a 2D en face image in FAF and a 3D volume scan in OCT that are not registered (compare Figure 1)). Methods for data registration in between these modalities are not available in clinical practice and only little established in scientific literature.

Previous works have proposed several methods for the registration of FAF and other en face imaging modalities [8,9,10,11,12,13,14,15]. Similarly, efforts have been made to visualize OCT labels, e.g., by projecting them onto the accompanying en face infrared (IR) image, where B-scan positions are marked by the OCT device [16,17] or a joint reference space [18]. In [19] an en face image is created by reducing the OCT volume. Still, neither kind of approach alone allows registration between OCT and FAF findings.

Previous work approaching OCT to en face fundus image registration relied on blood vessel segmentation [20,21,22,23,24,25,26]. Recent work on OCT to en face fluorescein angiography (FAG) registration used scanning laser ophthalmoscopy (SLO) as an intermediate image modality but still required blood vessel segmentation [27,28]. Our proposed pipeline is similar in that we also utilize an intermediate image modality for registration (IR in our case) but different in that we do not require explicit blood vessel segmentation, which might be sensitive to image quality [29] and structural damages due to progression of CSCR.

For our work, we utilized data from the University Eye Clinic of Kiel, Germany, a tertiary care center that is specialized on CSCR patients and has a unique large database of over 300 long-term CSCR disease courses with a median follow-up of 2.5 years.

Given this database and modern image registration and computation algorithms, our contribution is twofold: (1) We propose a visualisation pipeline capable of registering OCT and FAF images and their labels with high accuracy. (2) To show the utility of our pipeline, we conduct a pilot study regarding disease progression on 12 representative CSCR cases. Using the registered labels from our pipeline, we can analyze the spatial and temporal relationship between SRF and HF. Contrary to the common hypothesis that SRF precedes HF [30,31], our first clinical interpretation of the results suggests that, in regard to chronological order of the pathologies, two disease patterns can be identified: SRF preceding HF and vice versa.

We are confident that our contributions lay most of the technical groundwork for a large study to establish the chronological relationship between HF and SRF formation. The findings from such a study could not only help in understanding these biomarkers and their relevance but also might be used to categorize them into different CSCR subvarieties.

## 2. Materials and Method

### 2.1. Materials

All of our data were acquired with institutional review board approval from the University Eye Clinic of Kiel, Germany. From an existing dataset of 326 patients with CSCR from 2003 to 2020, patients were selected for a retrospective study with a reliable diagnosis of chronically recurrent CSCR, at least three visits and a long term course of the disease (minimum 2 years). The exclusion criteria were an uncertain diagnosis of CSCR and acute forms of CSCR.

In this work, altogether, we used data from 21 of these 326 patients (see Table 1). For these 21 patient, relevant data were collected over an average timespan of 5.5 years (range from 0 to 11 years) with an average of 6.5 (range from 1 to 12) visits per patient and eye. The main body of our data consists of 186 triples of OCT volumes, IR images and FAF images. Triples were taken on the same date of the same eye. OCT volumes were acquired with a Heidelberg Spectralis OCT device (super luminescence diode, average wavelength 870 nm), Heidelberg, Germany, and contain 25 B-scans with Field of View (FoV) 6×6 mm (6 µm/px horizontal and 4 µm/px vertical resolution; distance between B-scans 250 µm). FAF and IR images were both taken with a FoV of 30° and an average resolution of 11.3 µm/px. For all FAF images, expert annotations labeling HF were available. For 162 OCT volumes, expert annotations labeling SRF were available. For the remaining 24 volumes, SRF labels can be predicted with our proposed segmentation network (see Section 2.2.1).

Two components in our pipeline required further training data. Our segmentation network was trained on additional annotated OCT volumes, for which no corresponding FAF images were available. Our utilized registration network (see Section 2.2.4) was trained on additional FAF, fluorescein angiography (FAG) and fundus images [8]. The subsets of our data as used for training and different evaluations are given in Table 1. Details regarding relevant metadata of those subsets are given in the according sections.

### 2.2. Technical Methods

**Figure 2:**
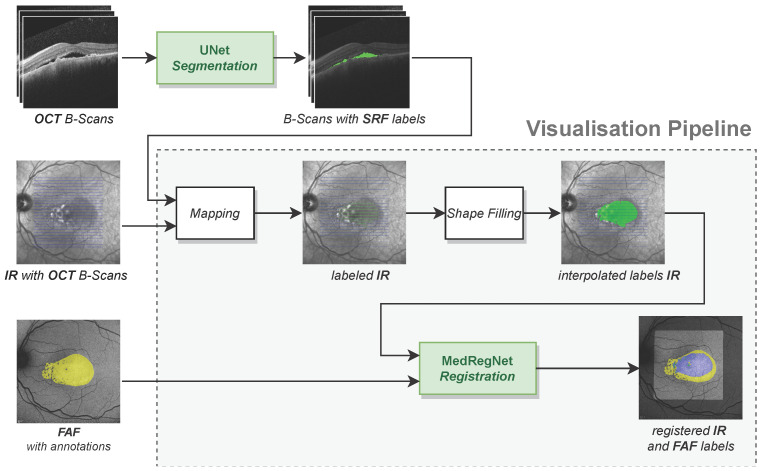
Our visualisation pipeline maps labels from OCT scans onto FAF images. On OCT B-scans for which we have no expert annotations, we utilized an U-Net-like segmentation model to predict SRF labels.

Our visualisation pipeline as depicted in Figure 2 takes as input annotated B-scans from an OCT, the corresponding IR image with the orthogonal position of each B-scan and an FAF image from the same eye. The output is a projection of OCT segmentations onto the FAF image, where label expansion in the gaps between the B-scans has been approximated by shape filling. In cases where no SRF expert annotations for the OCTs are available (compare Table 1), we used a segmentation network to predict SRF labels.

Our visualisation pipeline itself consists of the components: (1) the mapping function for projecting segmentations from OCT B-scans onto the corresponding IR image, (2) the shape filling algorithm to interpolate segmentations in the gaps between the B-scans, and (3) the MedRegNet registration module [8] to register the IR image and its segmentations onto the FAF image. Each component including the segmentation network is explained in the following subsections. Implementations of all components are given in the Supplementary Materials.

#### 2.2.1. OCT Segmentation Network

The segmentation network for prediction of SRF labels in OCT B-scans was inspired by the U-Net [32] and takes two inputs: the OCT B-scan to be segmented and the corresponding segmentation of the retina as given by the OCT imaging device. The encoding part of the network consists of four convolutional blocks followed by maximum pooling. Each block is composed of two convolutional layers with filter size 3×3, batch normalization and ReLU activation. To allow for different processing of the different input modalities, we do not pass B-scan and retina segmentation as a two-channel input but use two separate convolutional blocks at the first level of the network [33]. The resulting feature maps are concatenated after the first maximum pooling. The decoding branch mirrors the encoding branch and is connected to the encoder via skip connections on all levels. Upsampling is achieved with nearest neighbor interpolation. The output of the network after a final Sigmoid layer is the segmentation of SRF and pigment epithelial detachment (PED).

The network was trained on a NVIDIA DGX A100 system using one GPU for 300 epochs on 137 OCT images from 16 CSCR patients in our dataset (compare Table 1) with Adam optimization [34] and a constant learning rate of $1\times 10^{-4}$. As loss function, we used a generalized Dice loss [35] that compares predicted labels to manual annotations of SRF and PED.

#### 2.2.2. OCT to IR Mapping

**Figure 3:**
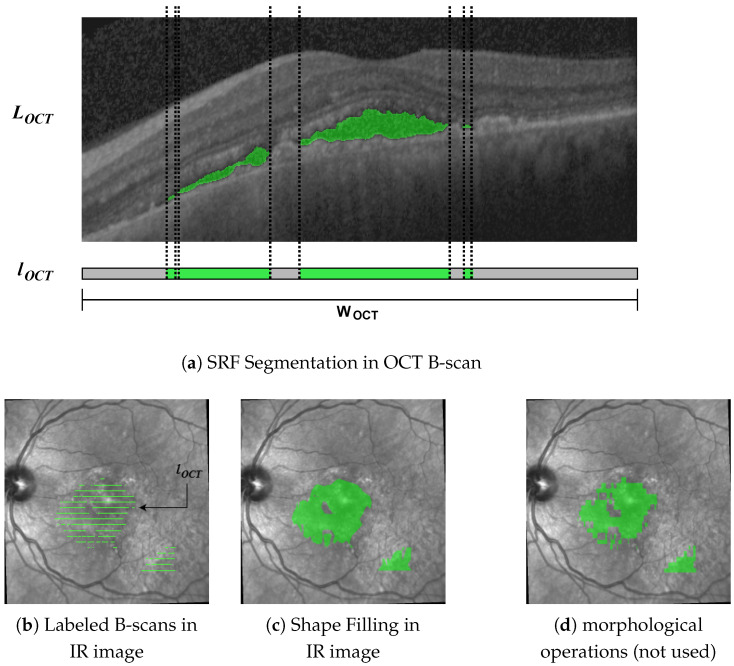
Segmentation masks throughout the different steps in our pipeline. We projected segmentations in the OCT B-scans (**a**) onto the IR image (**b**). To close the gaps between the B-scans, we used shape interpolation (**c**), which yielded better results than morphological dilation and closing (**d**).

In the first step, we mapped the labels from the B-scans onto the IR image. Given a B-scan of size $W_{OCT}\times H_{OCT}$, we denoted the corresponding segmentation mask of the same size as $L_{OCT}\in {\left\{0,1\right\}}^{W_{OCT}\times H_{OCT}}$. From this, we constructed a vector $l_{OCT}=\left[l_{1},\cdots ,l_{W_{OCT}}\right]\in {\left\{0,1\right\}}^{W_{OCT}}$, where for $1\leq i\leq W_{OCT}$, the entry $l_{i}$ is 1 if in column *i* of $L_{OCT}$ at least one pixel has value 1 and 0 else (see Figure 3a).

From metadata, we know the position and width *W* of each B-scan in the IR image; hence, we stretch or shrink $l_{OCT}$ to size *W* by nearest-neighbor interpolation before aligning the resulting vector with the B-scan position in the IR image. Doing this for all B-scans yields segmentation masks as in Figure 3b.

#### 2.2.3. Shape Filling

From Figure 3b, the gaps in the IR image between the labeled B-scans become apparent. Szeskin et al. [16] used morphological dilation and closing operations with experimentally determined parameters for closing the gaps. We found that this approach does not work well in our case, where OCT slices only number 25 of them compared with the 40–80 in [16] (compare Figure 3d). Instead, we used a shape-based interpolation method, as in [36], though simplified for use in 2D.

In the IR image each labeled B-scan is a binary label vector $l=\left[l_{1},l_{2},\ldots ,l_{W}\right]\in {\left\{0,1\right\}}^{W}$ with *W* entries, where 1 denotes the presence and 0 is the absence of the label at this pixel. From *l*, we retrieved a binary contour vector $c=\left[c_{1},\ldots ,c_{W}\right]\in {\left\{0,1\right\}}^{W}$, where

(1) $$c_{i}=\left\{\begin{array}{cc} 1, & \mathrm{if}\;l_{i}=1\;\mathrm{and}\;\left(l_{i-1}=0\;\mathrm{or}\;l_{i+1}=0\right)\\ 0, & \mathrm{else} \end{array}\right.$$

for 1≤i≤W. From *c*, we can calculate a distance vector $d=\left[d_{1},\ldots ,d_{W}\right]\in Z^{W}$, where

(2) $$d_{i}=\left\{\begin{array}{cc} \min_{c_{j}\in c,c_{j}=1}\left|i-j\right| & \mathrm{if}\;l_{i}=1\\ -\min_{c_{j}\in c,c_{j}=1}\left|i-j\right|, & \mathrm{else} \end{array}\right.$$

denotes the distance to the closest contour pixel in that B-scan. This distance is positive for label pixels and negative for non-label pixels. Between adjacent B-scans, those distance vectors $d^{1}$, $d^{2}$ are then linearly interpolated, such that a pixel at relative distance 0<f<1 between them is assigned value $d_{i}^{1,2}\left(f\right)=f\cdot d_{i}^{1}+\left(1-f\right)\cdot d_{i}^{2}$. Finally, pixels with an interpolated distance >0 are assigned as belonging to the label. The resulting segmentation in the IR images is shown in Figure 3c.

#### 2.2.4. IR to FAF Registration

**Figure 4:**
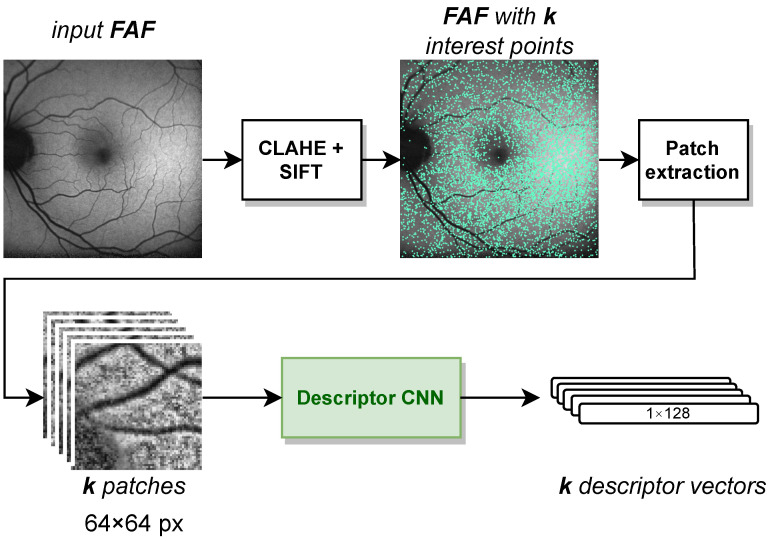
The steps inside the MedRegNet descriptor module [8]. Image contrast was improved with CLAHE [41]. Processing is identical for IR images.

To project the interpolated labels onto the FAF image, we need to register the IR image with the FAF image. For this, we used the feature-based multimodal MedRegNet descriptor module [8], as depicted in Figure 4. MedRegNet utilizes established point-detector algorithms such as SIFT [37] to detect interest points in both images. Rectangular 64×64 px patches are extracted around each interest points. These patches serve as input to MedRegNet’s lightweight convolutional neural network (CNN), which outputs for each patch a l2-normalized descriptor vector $dsc\in {\left[-1,1\right]}^{128}$.

Similar descriptors were then matched using Lowes ratio test [37], where we only kept those descriptors dsc in the first image with $\frac{||dsc-dsc_{1}{\left|\right|}_{2}}{||dsc-dsc_{2}{\left|\right|}_{2}}<\mu$. Here, $dsc_{1}$, $dsc_{2}$ were the first and second closest descriptors to dsc in the second image, respectively. From the remaining matches, we recovered a homography transformation from the first to the second images with RANSAC [38].

For our usecase, we utilized the multi- and monomodally pre-trained MedRegNet models of [8] without further training. We referred to the latter as MedRegNet${}_{mono}$. The multimodal MedRegNet as detailed in [8] was trained with SIFT points on the three image modalities FAF, fluorescein angiography (FAG) and fundus. MedRegNet${}_{mono}$ was trained with SIFT points on the public fundus images of [39,40]. Both sets of training were performed on a NVIDIA Titan Xp GPU and an Intel Core i7-4790K CPU with 4.00 GHz.

## 3. Technical Results

### 3.1. Segmentation Network

We evaluated the OCT segmentation network on 53 test images of 5 patients (compare Table 1) using the established [42] Dice score $DCS=\frac{2TP}{2TP+FP+FN}$ [43,44], where TP, FP and FN denote the true positive, false positive and false negative segmented pixels, respectively. For SRF segmentation, the network achieves a mean Dice score of 0.853 on B-scans containing SRF in the manual ground truth and outputs false positives in only 16 out of 958 B-scans containing no SRF. The SRF detection rate is 97.0% (385 out of 397 SRF-containing B-scans correctly detected). Probably due to imbalanced training data, the detection rate of PED is inferior at 43.9%, resulting in a mean Dice score of only 0.255. False positives are again seldom, with 23 out of 1232 B-scans containing erroneously predicted PED. For the visualisation pipeline, we only used the very reliable SRF segmentations.

**Figure 5:**
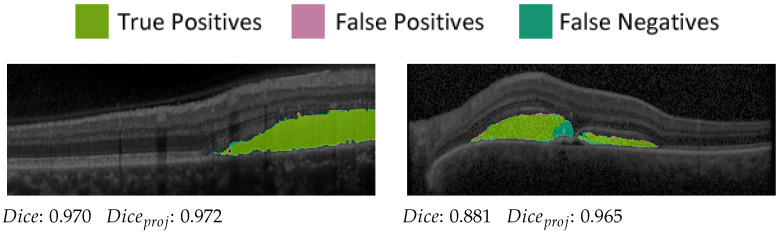
Two predictions and correspondingscores from the OCT evaluation set.

Furthermore, projection accuracy in our pipeline depends not on correct segmentation in the OCT volume itself but rather on correct segmentation in regard to the en face plane. Hence, to prove the suitability of the SRF segmentations for our visualisation pipeline, we projected ground-truth and predicted segmentation onto the en face plane. Pixels in the projection image were assigned a value of one if at least one voxel was segmented in the corresponding A-scan and zero otherwise. The Dice score of the projection images (compare Figure 5) was then calculated and averaged over all available examinations. We achieved a projected Dice${}_{proj}$ score of 0.897, which highlights the reliability of the SRF segmentations in the en face plane.

### 3.2. Registration Network

**Figure 6:**
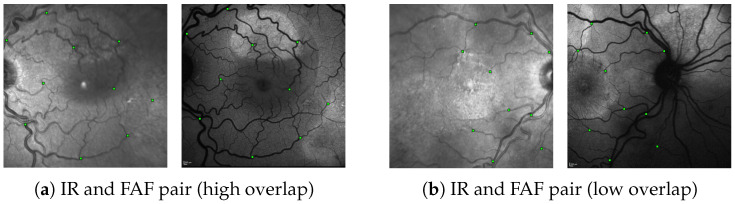
Two annotated IR↔FAF image pairs as encountered in our dataset. (**a**) An example with high spatial overlap and (**b**) and example with little spatial overlap. The green squares denote the 10 control-point pairs in each image pair used for evaluation (images are best viewed zoomed in).

Following the procedures and the registration error (RE) metric in [45], we evaluated the registration performance for each image-pair by utilizing a set of annotated 10 control-point pairs, denoting the same points in both images (see Figure 6). Given a proposed transformation *H* between the images, we projected control points from the first image onto the second image and calculated the RE as the mean euclidean distance of all control-point pairs. An image pair was considered successfully registered if enough points were matched to reconstruct some *H* and if, for that *H*, RE does not exceed a threshold. As in [45], we determined the percentage of successfully registered images for thresholds between 0 and 25 px and reported the resulting Area under Curve (AuC).

Regarding the evaluation data, since our IR and FAF images stem from the same pool of data as the FAF, FAG and fundus images used to train MedRegNet, we made sure to only include images from patients outside MedRegNet’s train dataset. With this, we show the registration performance on 34 same-date IR↔FAF image pairs from nine patients containing left and right eyes (compare Table 1). For each patient, if available, the images were chosen from the first and last date to cover different stages in CSCR progression.

**Figure 7:**
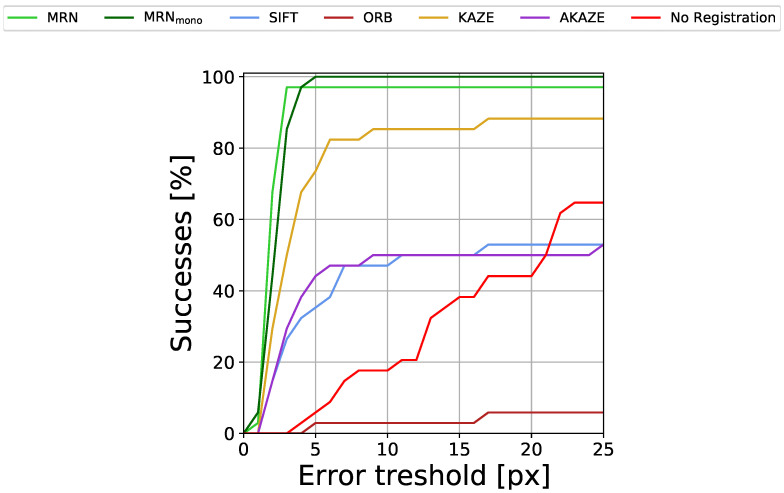
Percentage of successfully registered images for different registration error (RE) thresholds.

To show that IR↔FAF registration is no trivial task, we also present the registration performance of the common interest pointer detector and descriptor algorithms SIFT [37], ORB [46], KAZE [47] and AKAZE [48] as well as scores for not carrying out any registration at all. For each method, we chose the 0.5<μ<1 with the respective highest AuC to avoid selection bias. The results are given in Table 2 and Figure 7.

Evidently, both versions of MedRegNet, despite never having seen IR images during training (and MedRegNet${}_{mono}$ not having seen FAF images either) perform best with over 0.90 and 0.91 AuC. MedRegNet is able to register all but one image pair with RE <3 px, while MedRegNet${}_{mono}$ is able to register all images with RE <5 px. From the baselines, only KAZE performs with some accuracy, reaching 0.76 AuC, though it too fails to successfully register multiple image pairs even for the largest error threshold.

## 4. Technical Discussion

Regarding the technical implementation, we considered each of our single components to be of high precision: our network for SRF segmentation in OCT scans reaches a Dice score of 0.853. This score is similar to comparable state-of-the-art architectures predicting SRF for CSCR (0.910 [49]) or more general for macular edema (0.845 [50], 0.75 [51], 0.958 [52]). Our projected en face Dice score as relevant for our usecase is still higher with 0.897. While the shape filling algorithm cannot be evaluated quantitatively due to the lack of dense SRF annotations in the en face plane, visual inspections show the resulting shapes to appear more natural than previous approaches using morphological operations [16].

Both MedRegNet registration networks on our multimodal data outperform established off-the-shelf detector/descriptor algorithms (0.913 AuC and 0.902 vs. 0.760 AuC for KAZE [47]). Though MedRegNet${}_{mono}$ performs slightly better than MedRegNet, it might be less robust in low overlap images due to lower *TP* matches. The good performance of MedRegNet${}_{mono}$ despite never having seen IR or FAF images in training might be explained by (1) blood vessels appearing darker than the surrounding tissue in IR and FAF images similar to the fundus images that MedRegNet${}_{mono}$ was trained on and (2) the brightness and contrast augmentation during training.

It is also worth mentioning that, while some of the aforementioned single components in our pipeline are specialized for their respective modalities, the general layout of our pipeline is applicable for registration between sparse 3D cross-section and en face images as long as a spatial alignment from the cross-sectional scans to some en face modality (e.g., IR [16,17], SLO [27,28], reduced OCT volume [19]) can be established.

## 5. Medical Analysis

Our medical analysis was conducted in the context of the often-cited hypothesis that FAF changes depict chronic damage induced by SRF presence, i.e., that SRF formation always precedes HF development [30,31]. However, this has not conclusively been proven. Since chronic RPE alterations are thought to play an important role in disease pathophysiology, it could also be hypothesized that FAF changes precede SRF formation. Therefore, we wanted to challenge hypotheses on the chronological order.

### 5.1. Visualisation Method

One important distinction between both pathologies lies in their temporal consistency. While HF, once appearing, is generally observed to stay, SRF fluctuates heavily over time. This introduces temporal complexity to the analysis: a straightforward comparison of SRF and HF on the same date does not suffice, as SRF that could have influenced HF progression might no longer be visible at the current date—that is, if it has been captured at all, which due to intervals in the patient visits is not guaranteed.

**Figure 8:**
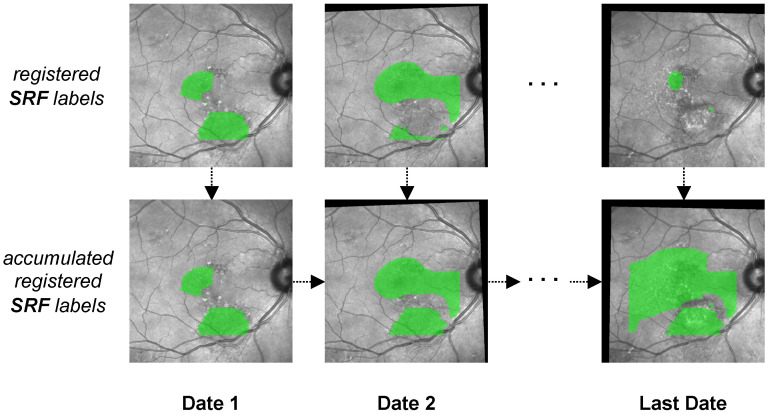
The process of generating accumulated SRF labels (**bottom** row) from single SRF labels (**top** row) over multiple dates. In the accumulated SRF labels, each pixel that at some point up to the current date had been observed as being occupied by SRF is colored in green. The process is identical for HF labels.

To account for the temporal component in our analysis, we chose the following method: Instead of only visualizing labels from the same date, we chronologically accumulated segmentations over time (see Figure 8). Hence, in our visualisation for a certain date, we labeled every pixel that had ever been occupied by either pathology up to this date. We accomplished this by regarding the initial FAF and IR image as anchors. Every following FAF and IR image together with their segmentation masks was first registered with MedRegNet onto their respective anchor, after which we used the projection of our visualisation pipeline to combine both into a single image. For each pixel where HF and SRF biomarkers overlap, we analyzed if SRF was present first, HF was present first, or the chronological order could not be established because both biomarkers were either present at baseline or appeared in the same follow-up on previously unoccupied space.

To analyze the chronological relationship between SRF in OCT scans and to diffuse HF in FAF images, we chose representative cases of chronic CSCR from our dataset where SRF and HF were both present and at least one entity was progressing. The result is an exemplary subset with 17 eyes of 12 patients (compare Table 1). Patients in this subset have a mean age of 49.8 years (range from 39 to 66 years) and 83.3% were male (10 of 12 patients). The mean disease course captured in our clinic was 5.25 years (range from 2 to 10 years) with an average of 7 visits (range from 3 to 12 visits, OCT and FAF performed in all visits).

## 6. Medical Discussion

Using our visualisation pipeline and the aforementioned accumulated visualisation from Section 5.1 on our subset of 12 patients and 17 eyes, we analyzed the chronology between SRF and HF. Indeed, two different patterns of disease course can be found in our pilot study: patients in which SRF seems to precede HF formation (pattern A) and vice versa (pattern B). We attributed all patients to either group; three eyes where no decisive order could be found were excluded. Table 3 shows these findings for the last follow-up point of all analyzed patients.

From the data in this table, we proposed the possibility of two CSCR disease distinct patterns and noted the following: HF development in an area of the retina which at the current or a previous time was only occupied by SRF (pattern A) gives a strong indication that SRF does indeed precede the development of HF. The inverted implication in cases where HF precedes SRF (pattern B), however, is not generally applicable, since in this area, SRF could have occurred and already been resolved in a time span when no picture was taken.

**Figure A1:**
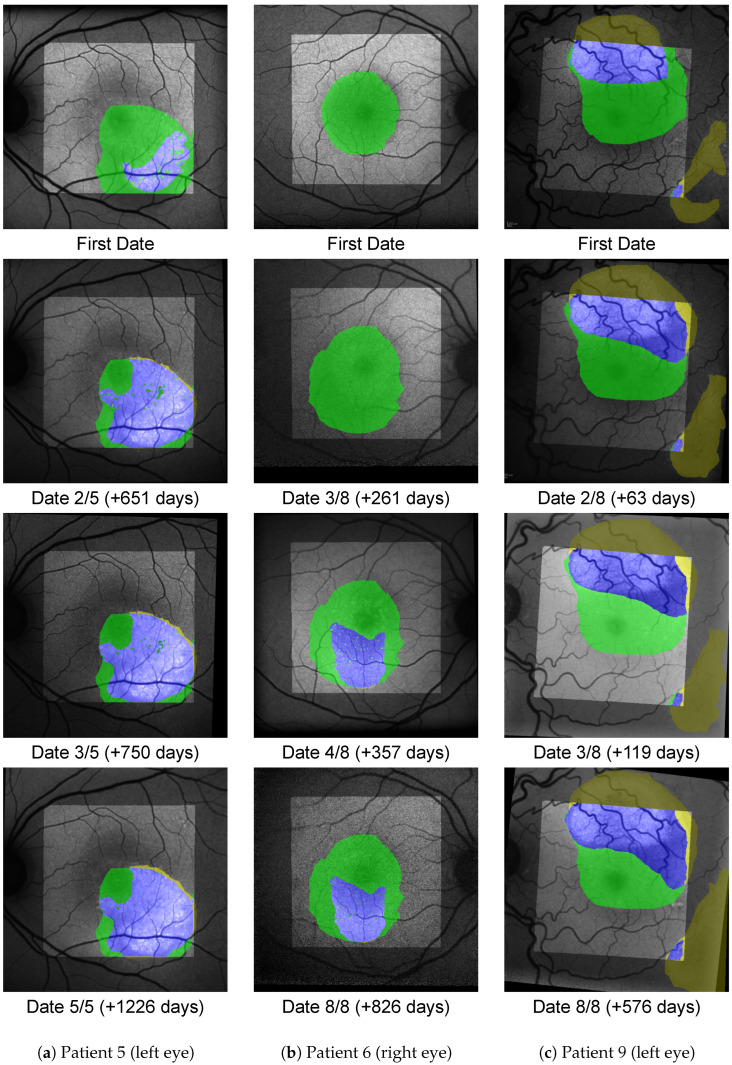
Accumulated areas of SRF (green), diffuse HF (yellow) and overlap (blue) for three eyes from pattern A, where SRF precedes HF. The background image is the FAF image for the given date registered to fit the FoV of the FAF image from the first date. The bright area shows the accumulated FoV from the OCT B-scans, i.e., outside of it no green or blue label can appear.

**Figure A2:**
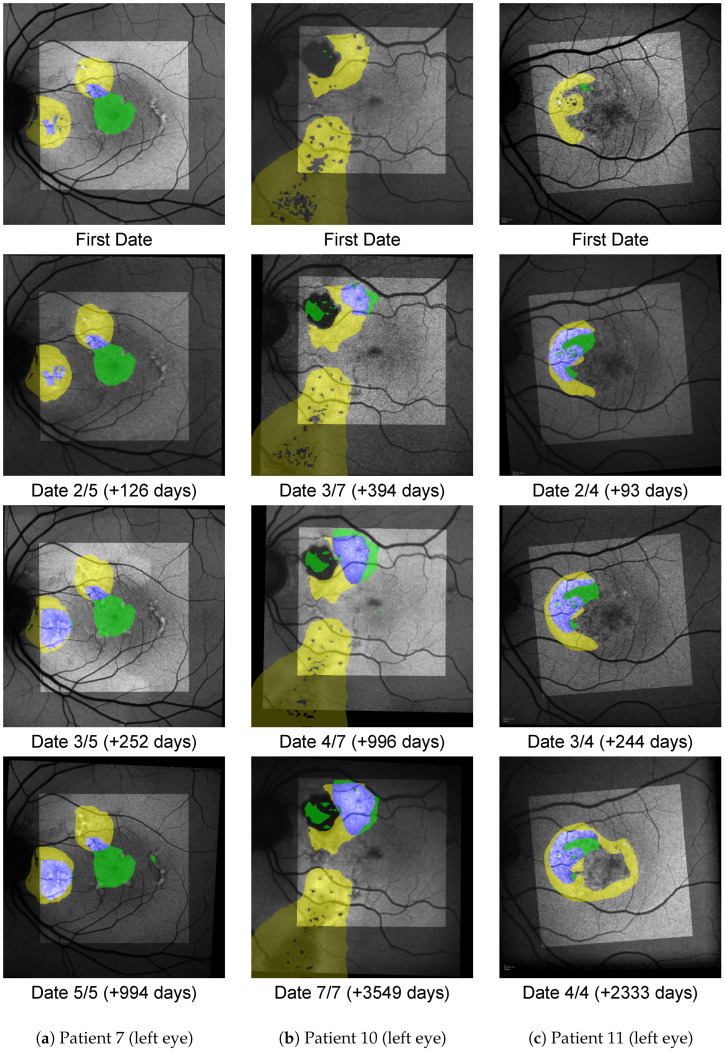
Shown here are progressions from pattern B, where HF precedes SRF.

**Figure A3:**
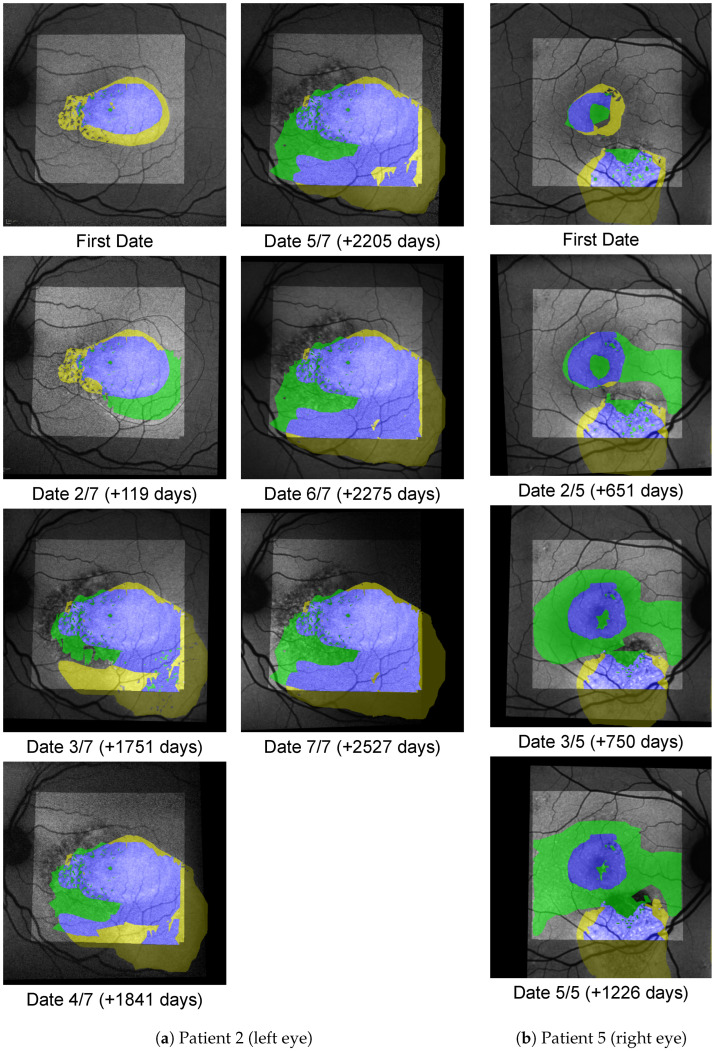
Shown here are progression from the excluded group, where no chronology can be established.

Whereas pattern A has previously been hypothesized [30,31], confirming pattern B in addition could be of high clinical relevance: From Table 3 patients with pattern A seem to take a prognostically more favorable course with regard to visual acuity (VA) development (measured in logMAR, meaning smaller value is higher VA). The mean VA for pattern A was 0.13 (median 0; range from 0 to 0.6) and 0.28 for pattern B (median 0.22; range from −0.1 to 0.89). Exemplary visualisations of representative disease courses can be found in Figure A1, Figure A2 and Figure A3.

Due to the clinical relevance of confirming (1) the existence especially of pattern B in chronic CSCR and if so (2) differences in VA development between the patterns, an application of our visualisation pipeline for larger datasets is planned. Furthermore, prospective studies could significantly enhance the data quality. In addition, with prospective studies and frequent OCT follow-up, the probability of a missing SRF occurrence would shrink.

For the moment, our pilot study confirms that using accumulated pathologies from SRF and HF segmentations registered with our pipeline is a useful tool in monitoring and evaluating CSCR disease progression. To the best of our knowledge, we are the first to set into context spatial and temporal development of SRF and HF in this way.

We also direct attention to the potential of automated CSCR pattern detection: While the presented grouping into the two patterns was performed by medical experts using both the visualisations and the area percentages presented in Table 3, it currently appears that for most courses comparing area percentages could suffice. Especially in the patients with pattern A, there seem to be no areas where HF precedes (0 to 1%, the 1% possibly due to segmentation errors), which allows for easy automated detection. We believe that our approach could be valuable in supporting clinicians by automatically pointing out recognized disease patterns.

## 7. Limitations

We identified the following technical limitation: while our automated segmentation of SRF in OCT scans reaches high Dice scores, we currently do not possess a mechanism to automatically segment HF in FAF images. If we did, our visualisation pipeline could operate completely on raw OCT and FAF data, i.e., on the image data as it is already acquired in current medical practice anyway. Hence, the possibility would arise to utilize our pipeline even during the appointment in which these images are taken.

The lack of an available HF segmentation algorithm can be explained by a limited amount of available annotated FAF data. As such, only few previous works have approached leakage segmentation in FAF images [53,54,55].

Regarding the limitations of our medical analysis, we note that our presented results are a pilot analysis on a small dataset (17 eyes from 12 chronic CSCR patients). Furthermore, the study was performed retrospectively with no fixed follow-up dates. Therefore, we observe long gaps between visits with no imaging in between. Hence, while we propose the possibility of different disease patterns, larger follow-up studies are required to confirm our propositions and to allow for statistically significant observations.

Furthermore, SRF and HF annotations are currently performed only by one expert. In the future, annotations from multiple experts would be desirable to analyze inter-observer reliability.

## 8. Conclusions

Our visualisation pipeline is capable of precise and robust projections of optical coherence tomography (OCT) segmentations onto en face retinal image modalities. This closes a gap in the medical image analysis of eye diseases and allows for joint assessment of pathologies from many different sources.

As an exemplary use of our pipeline, we analyzed the highly relevant relationship between subretinal fluids development (SRF) and diffuse hyperfluorescence (HF) progression in 17 eyes of 12 patients diagnosed with central serous chorioretinopathy. The results showed that, in most cases, one of two patterns could be observed: SRF preceding HF or vice versa. A large study on this topic is desirable. For such a study as well as in general clinical applications, our visualisation pipeline promises to be a valuable tool in analyzing spatial and temporal relationships between pathologies from OCTs and retinal imaging modalities.

## Figures and Tables

**Table 1:** We used image data from 21 CSCR patients: *seg. train* and *seg. eval* depict patients whose OCTs were used for training (Section 2.2.1) and evaluating (Section 3.1) the SRF segmentation network. *reg. train* depicts patients, from which FAF, FAG and fundus images were used to train the registration model in [8]. *reg. eval* depicts patients whose IR and FAF data were used for evaluating the same registration model in our usecase (Section 3.2). *SRF prediction* depicts patients, for which at least part of their SRF was predicted with our segmentation network during medical analysis. *med. analysis* lists patients used in our medical analysis (Section 5.1). Please note that segmentation and registration are independent of each other and that the medical analysis is separate from the technical evaluation.

	Patient ID																				
Used in	1	2	3	4	5	6	7	8	9	10	11	12	13	14	15	16	17	18	19	20	21
*seg. train*	.	✓	✓	.	.	.	✓	✓	✓	✓	✓	✓	.	✓	✓	✓	✓	✓	✓	✓	✓
*seg. eval*	✓	.	.	✓	✓	✓	.	.	.	.	.	.	✓	.	.	.	.	.	.	.	.
*reg. train*	.	✓	.	✓	✓	.	.	.	.	.	✓	✓	.	.	.	.	✓	.	✓	.	.
*reg. eval*	.	.	✓	.	.	✓	✓	✓	✓	✓	.	.	.	.	✓	✓	.	✓	.	.	.
*SRF prediction*	.	.	.	.	.	.	✓	.	✓	.	.	.	.	.	.	.	.	.	.	.	.
*med. analysis*	✓	✓	✓	✓	✓	✓	✓	✓	✓	✓	✓	✓	.	.	.	.	.	.	.	.	.

**Table 2:** Registration results. MRN is MedRegNet. *TP* matches are the average number of correctly matched interest points, i.e., point pairs that after registration have an euclidean distance <5 px. The number in brackets shows the percentage of all matched points, which are TP. AuC is the Area under Curve for the plots shown in Figure 7.

Method	*TP* Matches		AuC
MRN	1715	(34%)	0.902
MRN${}_{mono}$	606	(13%)	0.913
SIFT	55	(1%)	0.434
ORB	29	(0.3%)	0.034
KAZE	205	(8%)	0.760
AKAZE	60	(3%)	0.438
No registration	-		0.288

**Table 3:** Temporal analysis of the relationship of SRF and HF progression. VA is visual acuity development measured in logMAR, meaning a smaller value is a higher VA. According visualisations are given in Figure A1, Figure A2 and Figure A3.

Patient ID and Eye	Follow Up Time (Years)	Area of HF/SRF Overlap	Area Where SRF Precedes	Area Where HF Precedes	Area Where Chronology Is Unknown	Last VA (logMAR)
Pattern A: SRF Precedes						
5 L	3.3	68k px	67%	0%	33%	0.1
6 R	2.3	30k px	100%	0%	0%	0.6
8 L	2.5	11k px	98%	0%	2%	0
9 L	6.4	74k px	40%	1%	59%	0
11 R	6.4	39k px	100%	0%	0%	0
Pattern B: HF Precedes						
1 R	2.6	105k px	50%	41%	9%	0.4
3 R	7.6	0.4k px	1%	46%	53%	−0.1
4 L	6.0	74k px	37%	63%	0%	0.2
7 L	2.7	16k px	5%	71%	24%	0.1
9 R	6.4	76k px	28%	52%	20%	0.9
10 L	9.7	20k px	24%	74%	1%	0.4
10 R	9.7	81k px	6%	73%	21%	0.2
11 L	6.4	15k px	14%	82%	4%	0.2
12 L	7.5	31k px	1%	72%	27%	0.2
Excluded: No chronological order						
1 L	2.6	85k px	4%	19%	77%	0.2
2 L	7.9	132k px	20%	44%	36%	0.8
5 R	3.3	55k px	11%	21%	68%	1.5

## Data Availability

The data are currently not publicly available.

## Supplementary Materials

The code of our pipeline components can be downloaded at: https: //www.mdpi.com/article/10.3390/diagnostics1010000/s1.

## Appendix A

This appendix shows, in Figure A1, Figure A2 and Figure A3, exemplary visualisations of representative disease courses.
