# Supplementary figures and images for: Chronological Registration of OCT and Autofluorescence Findings in CSCR: Two Distinct Patterns in Disease Course
Source: Diagnostics (Basel). 2022 Jul 22;12(8):1780. doi: 10.3390/diagnostics12081780 (PMC9332035; doi:10.3390/diagnostics12081780)

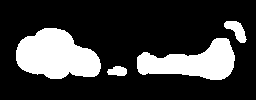

Supplement: Supplementary file 1 [file diagnostics-12-01780-s001.zip › MDPI_supplementary/shape_filling_code/data/FakeOCTsegs1.png]

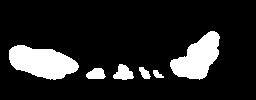

Supplement: Supplementary file 1 [file diagnostics-12-01780-s001.zip › MDPI_supplementary/shape_filling_code/data/FakeOCTsegs2.png]

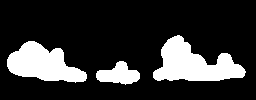

Supplement: Supplementary file 1 [file diagnostics-12-01780-s001.zip › MDPI_supplementary/shape_filling_code/data/FakeOCTsegs3.png]

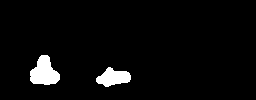

Supplement: Supplementary file 1 [file diagnostics-12-01780-s001.zip › MDPI_supplementary/shape_filling_code/data/FakeOCTsegs4.png]

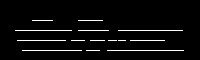

Supplement: Supplementary file 1 [file diagnostics-12-01780-s001.zip › MDPI_supplementary/shape_filling_code/out/IR_seg_line_mask.png]

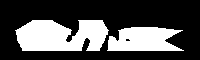

Supplement: Supplementary file 1 [file diagnostics-12-01780-s001.zip › MDPI_supplementary/shape_filling_code/out/IR_seg_shape_filled_mask.png]
